# Bacterial Vaginosis vs. Aerobic Vaginitis: An Unresolved Conundrum?

**DOI:** 10.3390/biology15161393

**Published:** 2026-08-14

**Authors:** Lorenzo Agoni, Canio Martinelli, Francesco De Seta

**Affiliations:** 1 Unit of Obstetrics and Gynecology, Fondazione Poliambulanza Istituto Ospedaliero, 25124 Brescia, Italy; lorenzo.agoni@poliambulanza.it; 2 Sbarro Institute for Cancer Research and Molecular Medicine and Center of Biotechnology, College of Science and Technology, Temple University, Philadelphia, PA 19122, USA; canio.martinelli@shro.org; 3 Unit of Obstetrics and Gynecology, Department of Human Pathology of Adult and Childhood “Gaetano Barresi”, University of Messina, 98124 Messina, Italy; 4 Department of Obstetrics and Gynecology, IRCCS San Raffaele Scientific Institute, 20132 Milan, Italy

**Keywords:** bacterial vaginosis, aerobic vaginitis, vaginal dysbiosis, differential diagnosis, vaginal microbiome, mixed vaginitis, microscopy, vaginitis guidelines

## Abstract

Bacterial vaginosis (BV) and aerobic vaginitis (AV) are usually described as distinct forms of vaginal dysbiosis. BV is characterized by depletion of lactobacilli, overgrowth of anaerobic microorganisms, and biofilm formation, whereas AV is associated with inflammatory changes, epithelial disruption, and predominance of aerobic bacteria. Over the past two decades, this distinction has profoundly influenced the understanding, diagnosis, and management of vaginal disorders. Despite their apparent differences, the relationship between BV and AV remains incompletely understood. Growing evidence indicates that many women exhibit microbiological and microscopic patterns that cannot be readily classified within existing diagnostic frameworks. Intermediate Nugent scores, mixed vaginitis, transitional ecological states, post-treatment microbiota reconstitution, physiological hypoestrogenic conditions, and uncommon inflammatory patterns all challenge the traditional view of BV and AV as strictly separate entities. This narrative review examines the historical evolution of BV and AV concepts, compares their microbiological, immunological, and epithelial characteristics, and evaluates the diagnostic paradigms currently used to identify vaginal dysbiosis. The strengths and limitations of clinical and diagnostic approaches are discussed together with the extent to which contemporary international guidelines reflect current biological knowledge. Particular emphasis is placed on intermediate, mixed, and overlapping states that blur the boundaries between established diagnostic categories. The available evidence supports the clinical usefulness of distinguishing BV and AV as separate clinicopathological entities. However, it also indicates that vaginal ecosystem disturbances frequently extend beyond rigid dichotomous classifications. Current diagnostic categories remain valuable tools for clinical practice, yet they may only partially capture the complexity and dynamic nature of vaginal dysbiosis. Understanding how microbial communities, host responses, epithelial integrity, and physiological factors interact remains a major challenge for future research and clinical interpretation.

## Simple Summary

The vaginal environment is normally dominated by beneficial bacteria that help protect women from infection. When this balance changes, different types of vaginal disorders can develop. Two of the best-known conditions are bacterial vaginosis and aerobic vaginitis, which are usually diagnosed and treated as separate diseases because they have different microscopic features and different patterns of inflammation. However, not all women fit clearly into one of these two categories. In everyday clinical practice, many patients show mixed or intermediate findings that are difficult to classify using current diagnostic systems. Hormonal changes, recovery after treatment, and other biological factors may further complicate the interpretation of vaginal samples. This narrative review examines how current concepts of bacterial vaginosis and aerobic vaginitis have evolved, summarizes their similarities and differences, and discusses the limitations of existing diagnostic approaches. The available evidence suggests that vaginal disorders are often more biologically diverse than traditional classifications imply, highlighting the need for careful interpretation of microscopic findings within the broader clinical context.

## 1. Introduction

Abnormal vaginal discharge is one of the most frequent reasons for gynecological consultation worldwide and represents a major burden on women’s reproductive health [1]. Among the disorders associated with vaginal dysbiosis, bacterial vaginosis (BV) has received the greatest attention because of its high prevalence, long-recognized status, and its well-established association with adverse reproductive outcomes, including preterm birth, pelvic inflammatory disease, and an increased risk of sexually transmitted infections (STIs) [2,3,4]. As a result, BV has emerged as the primary conceptual framework for interpreting alterations of the vaginal microbiota in both clinical practice and research.

Conventionally, BV has been regarded as a polymicrobial dysbiosis characterized by depletion of *Lactobacillus* spp., overgrowth of anaerobic microorganisms, and, in many cases, the development of a structured biofilm adherent to the vaginal epithelium [5,6,7]. The introduction of the diagnostic criteria proposed by Amsel et al. [8] in 1983 and the Gram stain-based scoring system developed by Nugent et al. [9] in 1991 provided standardized diagnostic methods that still form the basis of routine clinical diagnosis as well as most epidemiological and therapeutic studies.

Not all women with vaginal dysbiosis symptoms, however, fulfill the diagnostic criteria for overt BV. In 2002, Donders et al. [10] described aerobic vaginitis (AV) as a distinct clinicopathological entity characterized by depletion of lactobacilli, predominance of aerobic microorganisms, prominent inflammation, and epithelial disruption [10]. Since then, AV has gained increasing recognition, particularly in European clinical practice, where it is now considered a significant cause of vaginitis [11]. Nevertheless, its nosological status remains controversial and is not widely accepted outside Europe, and its incorporation into international diagnostic guidelines has been inconsistent [12,13,14,15].

Despite the widespread availability of culture-based and molecular diagnostic techniques, microscopy continues to occupy a central role in the evaluation of vaginal dysbiosis. Gram-stained smears interpreted according to the Nugent scoring system remain the laboratory reference standard for BV, whereas wet mount microscopy interpreted using the Donders score allows the diagnosis and grading of AV [9,10]. This distinction highlights a major difference in the diagnostic philosophies underlying the two scoring systems: one primarily focused on microbial composition and the other incorporating both microbial and host-related microscopic features.

The advent of molecular technologies, including polymerase chain reaction (PCR), other nucleic acid amplification tests (NAATs), and next-generation sequencing (NGS), has further enhanced the understanding of the dynamics of the vaginal ecosystem, increasing both sensitivity and specificity. By combining all available techniques with longitudinal studies, the vaginal microbiota is now recognized as a dynamic ecological community influenced by hormonal status, age, sexual activity, host immunity, and numerous environmental factors [16,17,18]. However, intermediate, mixed, and transitional microbial states have emerged as far more common than previously appreciated, challenging the traditional boundaries between established diagnostic categories [19,20].

These observations have important implications for both clinical practice and research. Different diagnostic approaches may classify the same patient differently because they evaluate distinct biological dimensions. Consequently, diagnostic discrepancies may affect therapeutic decisions, interpretation of clinical outcomes, and the comparability of research findings. More fundamentally, they raise the question of whether current diagnostic classifications adequately reflect the biological complexity of vaginal dysbiosis encountered in everyday clinical practice.

This narrative review critically examines the historical evolution of the concepts of BV and AV, compares their microbiological and cytopathological features, evaluates the strengths and limitations of current diagnostic approaches, and discusses the growing evidence on the significance of intermediate, mixed, and transitional vaginal states. Finally, it considers whether the traditional distinction between BV and AV fully captures the complexity of vaginal dysbiosis or instead represents a pragmatic simplification of a far more intricate dynamic biological reality.

## 2. Literature Search Methodology

This manuscript was conducted as a narrative review aimed at providing an integrated overview of the historical evolution, pathophysiology, diagnostic approaches, and clinical interpretation of BVand AV. A non-systematic literature search was performed using PubMed/MEDLINE, and Scopus to identify relevant publications available up to June 2026.

Searches included combinations of the following keywords: bacterial vaginosis, aerobic vaginitis, vaginal dysbiosis, Nugent, Donders, vaginal microbiome, wet mount microscopy, Gram stain, cytolytic vaginosis, desquamative inflammatory vaginitis, *Lactobacillus*, biofilm, vaginal inflammation, and related terms. Additional relevant publications were identified through manual screening of the reference lists of selected articles.

Priority was given to landmark studies, original research articles, systematic reviews, clinical guidelines, and consensus documents that contributed substantially to the current understanding of BV and AV. When appropriate, older seminal publications were included because of their historical relevance to the development of current diagnostic concepts.

As this is a narrative review, no formal systematic review methodology, risk-of-bias assessment, or certainty-of-evidence grading (e.g., GRADE) was performed. Consequently, the conclusions presented should be interpreted as an evidence-informed narrative synthesis rather than as recommendations derived from a systematic review or meta-analysis.

## 3. Historical Evolution of BV and AV Concepts

### 3.1. From Nonspecific Vaginitis to Bacterial Vaginosis

The understanding of what was once termed “abnormal vaginal flora” has evolved profoundly over the past century, reflecting major advances in both microbiology and diagnostic methodology. Before the recognition of BV, the evaluation of women presenting with vaginal discharge relied almost exclusively on microscopy, which enabled the identification of the two best-established infectious causes of vaginitis at that time: vulvovaginal candidiasis and trichomoniasis. The demonstration of fungal *Candida* elements, hyphae/pseudohyphae or budding yeast cells”, or motile *Trichomonas vaginalis* protozoa on wet mount examination therefore constituted the cornerstone of etiological diagnosis [1].

However, a considerable proportion of symptomatic women exhibited neither fungal elements nor protozoa. In the absence of an identifiable pathogen, these cases were collectively classified as “nonspecific vaginitis,” a diagnosis of exclusion that reflected the limited understanding of vaginal microbial ecology until the mid-twentieth century [21].

A turning point came in 1955, when Gardner and Dukes described the association between a previously unrecognized microorganism, initially designated *Haemophilus vaginalis* and later reclassified as *Gardnerella vaginalis*, and a syndrome characterized by homogeneous vaginal discharge in the absence of detectable inflammation [22]. The condition subsequently became known as “*Gardnerella* vaginitis” [23].

As knowledge of the vaginal microbiota expanded, it became evident that this syndrome was not attributable to a single pathogen but rather to a profound ecological disturbance characterized by depletion of lactobacilli and proliferation of multiple anaerobic bacteria [5]. The term “bacterial vaginosis” was therefore adopted to emphasize both its polymicrobial nature and its distinctive lack of a marked inflammatory response [24].

The first attempt to standardize the diagnosis of BV came in 1983, when Amsel et al. [8] proposed four clinical criteria based on vaginal discharge, elevated vaginal pH, a positive amine “whiff” test, and the presence of “clue cells” on microscopy. Although these criteria quickly gained widespread clinical acceptance because of their simplicity, they remained partly subjective and highly dependent on examiner experience.

Further standardization was achieved in 1991 with the development of the Gram stain-based system proposed by Nugent et al. [9], which remains the laboratory reference method for BV diagnosis to this day [9]. This scoring system quantifies bacterial morphotypes associated with lactobacilli, *Gardnerella*/*Bacteroides*, and curved Gram-variable rods, generating a composite score ranging from 0 to 10. Scores of 0–3 indicate normal flora, scores of 4–6 define the intermediate category, and scores of 7–10 are considered diagnostic of BV [9].

Despite its widespread adoption, the biological meaning of the intermediate category has remained uncertain. Initially conceived as a transitional stage between normal flora and BV, intermediate scores of 4–6 have subsequently been interpreted in multiple ways, including early BV, resolving disease, partial treatment response, abnormal flora, or simply grouped together with BV in epidemiological studies [25]. More importantly, this scoring system evaluates bacterial morphotypes alone and provides no information on inflammatory response, or epithelial integrity. Consequently, because it does not assess inflammation or epithelial alterations, women who fulfill diagnostic criteria for AV when evaluated with dedicated microscopic criteria may nevertheless be classified within the intermediate category when only the scoring system is applied [10,11]. Recognition of this limitation has become increasingly important as the complexity of vaginal dysbiosis has become more widely appreciated.

### 3.2. The Emergence of Aerobic Vaginitis

While BV progressively became established as the dominant model of vaginal dysbiosis, clinicians continued to encounter symptomatic women whose clinical and microscopic findings were inconsistent with classical BV. These patients frequently presented with purulent discharge, burning, dyspareunia, elevated vaginal pH, and pronounced inflammatory mucosal changes despite the absence of the characteristic anaerobic pattern observed in BV [10].

In 2002, Donders et al. [10] formally introduced the concept of aerobic vaginitis, defining it as a distinct clinicopathological entity characterized by depletion of lactobacilli, predominance of aerobic microorganisms, marked inflammation, and frequent detection of parabasal epithelial cells, indicative of epithelial injury and accelerated epithelial turnover [10]. Unlike BV, AV was conceived not simply as a microbiological alteration but as an inflammatory disorder arising from the interaction between changes in the vaginal microbiota and the host response. Accordingly, AV represents a pathophysiological condition distinct from BV and therefore requires a dedicated interpretative framework.

To enable diagnosis, Donders et al. [10] developed a composite microscopic scoring system incorporating lactobacillary grade (LBG), characteristics of the background flora, leukocyte abundance, presence of toxic leukocytes, and parabasal epithelial cells [10,11]. By integrating microbial and host-related parameters, this approach marked a conceptual shift away from purely microbiological classifications toward a broader microscopic assessment of vaginal health.

Since its original description, AV has gained increasing recognition, particularly among European clinicians and investigators [26]. Nevertheless, important questions remain unresolved. Its reported prevalence varies substantially among studies, diagnostic thresholds have not been universally standardized, and its relationship with other previously identified inflammatory conditions, particularly desquamative inflammatory vaginitis (DIV), remains a matter of debate [27,28].

These frameworks should therefore be regarded as complementary tools capturing different dominant pathological vaginal conditions.

### 3.3. The Vaginal Microbiome Era

The introduction of culture-independent molecular technologies, including PCR, NAATs, and NGS has fundamentally reshaped current understanding of the vaginal ecosystem [16]. These approaches have demonstrated that the healthy vaginal microbiota is generally dominated by *Lactobacillus* species, while also revealing substantial interpersonal, ethnic, and temporal variability [18].

Molecular investigations have further shown that vaginal dysbiosis extends well beyond the traditional dichotomy between health and BV. Rather than representing fixed microbial states, vaginal communities appear highly dynamic, with intermediate microbial configurations, mixed dysbiotic patterns, and continuous ecological fluctuations occurring in both symptomatic and asymptomatic women [17,18]. In parallel, these studies have established that *Gardnerella vaginalis* constitutes only one component of a complex polymicrobial ecosystem that also includes organisms such as *Fannyhessea vaginae* (formerly *Atopobium vaginae*), *Prevotella* spp., *Mobiluncus* spp., *Megasphaera* spp., and numerous other anaerobic bacteria [7,29].

Despite these important advances, molecular techniques have also highlighted their own limitations. These nucleic acid-based assays characterize microbial composition with remarkable sensitivity but provide no direct information regarding epithelial integrity, inflammatory activity, or host–microbe interactions, all of which remain fundamental for the diagnosis of AV and other inflammatory forms of vaginal dysbiosis [12]. Consequently, microscopy continues to occupy a central role in the evaluation of symptomatic women, particularly when inflammatory disorders are suspected.

**Figure 1:**
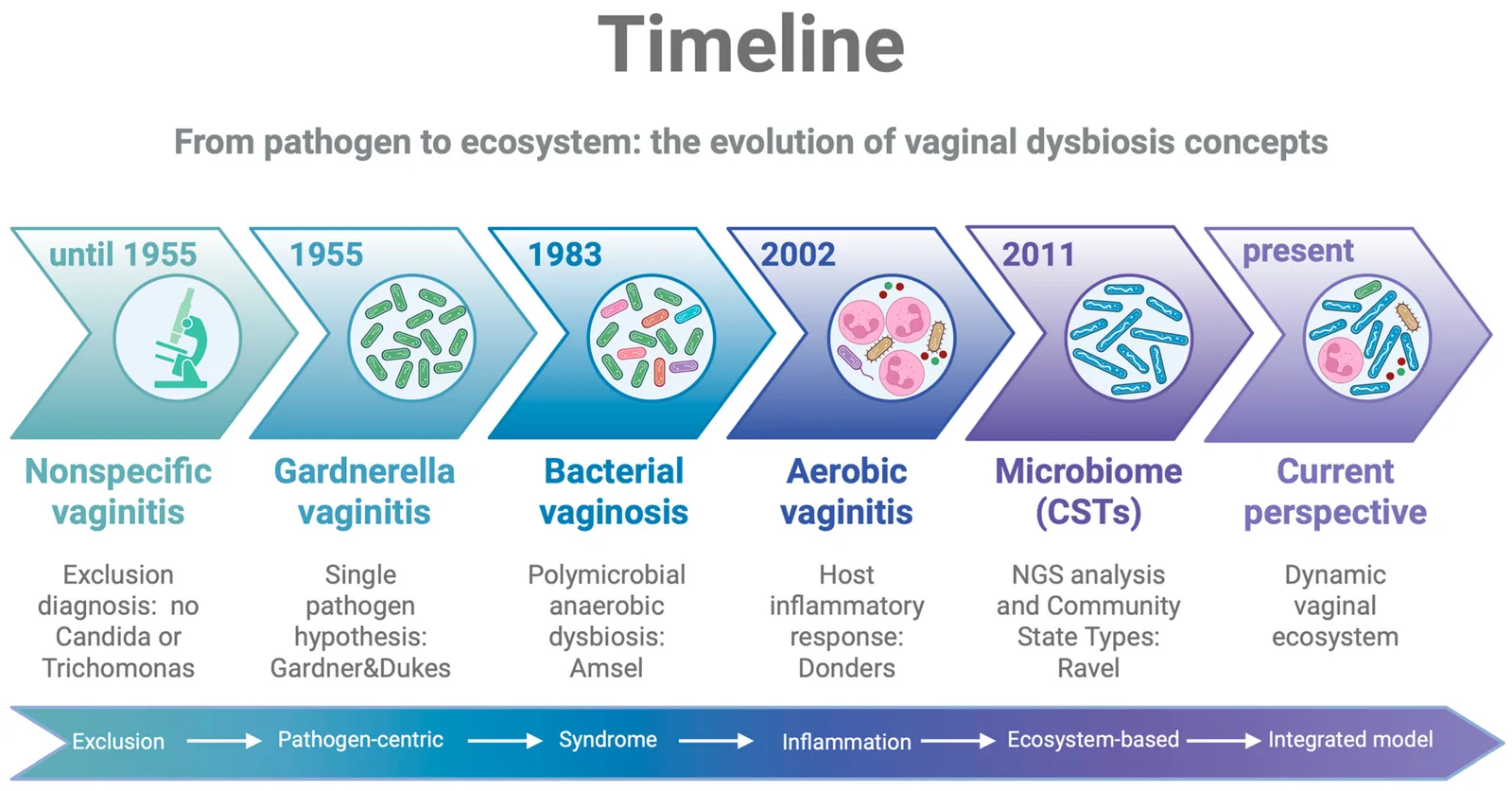
Historical evolution of concepts in vaginal dysbiosis. Timeline illustrating the historical progression of concepts related to vaginal dysbiosis, from nonspecific vaginitis to the establishment of bacterial vaginosis as a dominant diagnostic framework, the subsequent introduction of aerobic vaginitis as a distinct clinicopathological entity, and the transition toward microbiome-based interpretations of vaginal health. The figure highlights the progressive refinement and fragmentation of diagnostic paradigms over time. Created in https://BioRender.com.

Taken together, developments in molecular microbiology have reinforced the view that vaginal dysbiosis represents a dynamic ecological process rather than a series of rigid disease categories (Figure 1). At the same time, they have renewed the debate regarding the biological boundaries separating BV, AV, and the broad spectrum of intermediate or overlapping vaginal dysbiotic states.

## 4. BV and AV: Distinct Biological Entities?

### 4.1. Vaginal Microbiota Composition

BV and AV both arise in the setting of profound depletion of the normal *Lactobacillus*-dominated vaginal ecosystem. The microbial communities associated with these conditions, however, differ sufficiently to suggest that they represent distinct ecological patterns rather than simple variations of a single dysbiotic process [30,31].

BV is typically characterized by overgrowth of a diverse consortium of predominantly anaerobic microorganisms. Commonly identified organisms include *Gardnerella vaginalis* and *Fannyhessea vaginae* (formerly *Atopobium vaginae*), as well as *Prevotella* spp., *Mobiluncus* spp., *Megasphaera* spp., *Sneathia* spp., and numerous other anaerobes that are often difficult to culture using conventional microbiological methods [7,29,30,32]. Microscopic diagnosis of BV is based solely on a combination of *Lactobacillus* depletion and a dominance of *Gardnerella*/*Mobiluncus* morphotypes. Molecular investigations have further shown that BV-associated dysbiosis is highly polymicrobial and exhibits substantial interindividual variability [33]. Notably, these species may also be isolated in asymptomatic individuals, and their presence alone is not diagnostic of BV. According to the scoring system proposed by Nugent et al. [9], only the combination of depleted lactobacilli and dominance of *Gardnerella*/*Mobiluncus* morphotypes is diagnostic for BV [9].

AV is typically associated with a predominance of aerobic facultative microorganisms, most commonly *Escherichia coli*, *Enterococcus faecalis*, *Enterococcus faecium*, Group B *Streptococcus*, and *Staphylococcus* spp. [34]. Importantly, these organisms may also be recovered from vaginal microbial communities of asymptomatic women, indicating that microbial composition alone is insufficient to define AV [16,17]. Diagnosis depends on their association with inflammation, leukocyte recruitment, and epithelial desquamation, as defined by the Donders scoring system [10].

An additional ecological distinction deserves consideration. Many microorganisms frequently associated with AV are also common constituents of the gastrointestinal and perineal microbiota rather than dominant members of the healthy vaginal ecosystem. Their increased abundance in AV has therefore been hypothesized to reflect, at least in part, altered colonization dynamics involving adjacent reservoirs [35]. In contrast, BV is characterized by the expansion of complex anaerobic communities that are commonly associated to the vaginal ecosystem. From an ecological perspective, this difference supports the view that BV and AV represent distinct dysbiotic routes rather than sequential stages of the same disorder.

At the same time, the boundary between anaerobic and aerobic dysbiosis is not absolute. Mixed microbial patterns containing both BV-associated anaerobes and aerobic organisms are frequently encountered in clinical practice, complicating any rigid classification scheme [19,36,37].

### 4.2. Host Inflammatory Response

The most striking biological distinction between BV and AV lies not in the microorganisms themselves, but in the host response they elicit.

Classical BV has long been regarded as a predominantly non-inflammatory syndrome. Women with BV often show relatively few leukocytes on microscopy, and their symptoms are usually dominated by malodor and discharge rather than by signs of mucosal inflammation [10,29,38]. This apparent paradox—widespread bacterial presence despite the less pronounced neutrophilic inflammatory response typically observed in AV—has stimulated considerable investigation. Proposed mechanisms include immune evasion by BV-associated microorganisms, active modulation of mucosal immunity, induction of modulation of local immune response, and the protective effects of the polymicrobial biofilm that characterizes this condition [29,39].

AV, on the other hand, is characterized by abundant leukocytes, frequent toxic leukocytes, elevated concentrations of proinflammatory mediators, and clinically evident mucosal irritation. Burning, dyspareunia, erythema, and epithelial fragility are typical features of this syndrome [40].

Differences in microbial composition and host–microbe interactions may contribute to this divergence. Many AV-associated organisms are common inhabitants of the gastrointestinal and perineal microbiota [35], and their expansion within the vaginal environment may trigger a more vigorous mucosal inflammatory response than is typically observed in BV. Although this remains a hypothesis, it offers a plausible explanation for the markedly inflammatory phenotype observed in AV.

Viewed from this perspective, BV and AV differ not only in microbial composition but also in the nature of the host–microbe interaction they represent.

### 4.3. Biofilm Formation

Biofilm formation constitutes another major point of divergence between BV and AV.

In BV, a dense polymicrobial biofilm adherent to the vaginal epithelium is a well-established feature. *Gardnerella* spp. are thought to act as early colonizers and structural organizers, facilitating recruitment of additional anaerobic taxa to expand and stabilize the biofilm community [41,42]. This architecture contributes to treatment failure, persistence, and recurrence of BV after apparent initial treatment success.

The microscopic hallmark of this process is the presence of “clue cells”: epithelial cells heavily coated with an organized biofilm matrix, providing a morphological correlate of extensive bacterial adhesion and colonization [43,44].

Unlike BV, a consistently demonstrable polymicrobial epithelial biofilm has not been established in AV. Although several AV-associated organisms, including *Enterococcus faecalis* and *Staphylococcus aureus*, are capable of forming biofilms, current evidence does not support the existence of a characteristic, organized epithelial biofilm comparable to that observed in BV [45]. The absence of clue cells in AV therefore reflects not only a difference in microbial composition but also a fundamentally different spatial organization of host–microbe interactions.

These differences should not be interpreted as absolute. Transitional and mixed patterns do occur, particularly among women with Nugent scores of 4–6, where partial development of BV-associated epithelial colonization, evolving dysbiosis, or overlapping BV–AV features may coexist. Such observations reinforce the biological heterogeneity of vaginal ecosystem disturbances.

### 4.4. Limitations of Current Diagnostic Frameworks and the Intermediate Nugent Score Category

The widespread use of the Nugent scoring system has greatly improved standardization in BV research and clinical practice. Its conceptual simplicity, however, is also its principal limitation. By reducing vaginal microbiology to a simple morphotype-based score, the system compresses biologically heterogeneous states into a single numerical continuum. This limitation becomes particularly evident in the interpretation of intermediate scores (4–6).

The intermediate category is often viewed as a transitional stage between a healthy vaginal microbiome and BV. Increasing evidence suggests that this interpretation is overly simplistic. Rather than representing a single biological trajectory, intermediate scores likely encompass multiple ecological states, including early BV, inflammatory dysbiosis, and other biologically distinct vaginal conditions that cannot be distinguished by morphotype assessment alone.

The critical issue is not merely the lack of species-level resolution. Nugent scoring provides no information regarding inflammation, epithelial integrity, or host response. Consequently, fundamentally different biological conditions may converge within the same numerical range.

Importantly, the Nugent score was not designed to diagnose AV, and AV should therefore be assessed independently using dedicated microscopic criteria such as the Donders scoring system. Nevertheless, because the Nugent score is frequently applied as the sole microscopic classification method in both clinical practice and research, women who actually fulfil AV diagnostic criteria may be included within the biologically heterogeneous intermediate Nugent category when no dedicated AV assessment is performed.

**Figure 2:**
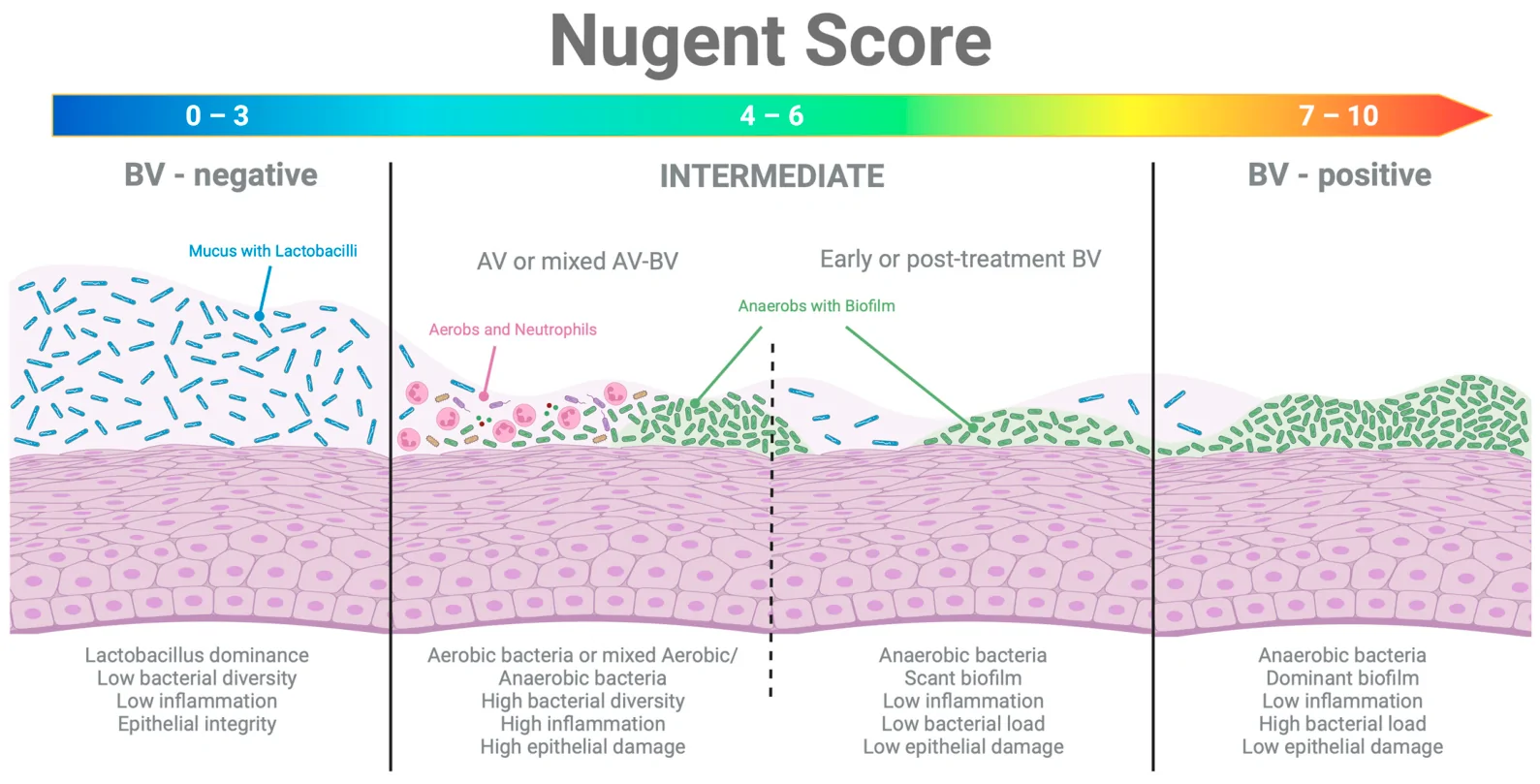
Diagnostic limitations of the Nugent scoring system and the intermediate (4–6) category. Schematic representation of the Nugent scoring system with emphasis on the intermediate range (scores 4–6), traditionally considered a transitional state between normal vaginal flora and bacterial vaginosis. The figure illustrates the biological heterogeneity underlying this category, which may include early or partial bacterial vaginosis, aerobic vaginitis-like states, post-antibiotic dysbiosis, hormone-dependent alterations, mixed anaerobic–aerobic dysbiosis, and low-grade indeterminate microbial imbalance. This heterogeneity highlights the limited specificity of the Nugent system in capturing non-classical vaginal dysbiosis patterns. Created in https://BioRender.com.

A score of 4–6 may therefore represent progression toward anaerobic BV, a relatively stable *Lactobacillus*-depleted equilibrium, or an inflammatory aerobic dysbiosis characterized by prominent immune activation. These scenarios are unlikely to be equivalent in terms of pathophysiology, prognosis, or therapeutic response, yet they are frequently grouped together under a single operational category (Figure 2).

The recognition of AV as a distinct inflammatory entity has made this limitation increasingly apparent. Accordingly, at least a subset of women classified as having intermediate Nugent flora may fulfill diagnostic criteria for AV, or for mixed BV–AV, when independently evaluated using dedicated AV microscopy.

### 4.5. Epithelial Alterations and Mucosal Damage

The vaginal epithelium is increasingly regarded as an active participant in mucosal immunity and ecosystem regulation, rather than merely a passive physical barrier or a site of microbial–epithelial interaction. Notably, the epithelial response to dysbiosis differs substantially between BV and AV.

In BV, epithelial architecture is usually preserved despite extensive bacterial colonization. Although BV-associated organisms can produce cytotoxic factors such as vaginolysin, clinically significant epithelial injury is generally limited, likely because biofilm organization restricts exposure of deeper tissues and because the associated inflammatory response is relatively attenuated.

AV, in contrast, is characterized by epithelial disruption, increased epithelial turnover, and desquamation. The presence of parabasal cells, particularly in moderate and severe cases, reflects accelerated regeneration and mucosal injury [10]. Clinically, erythema, petechiae, erosions, and contact bleeding are far more typical of AV than of BV. These changes are closely linked to intense local inflammation and leukocyte-mediated tissue damage.

As with other aspects of vaginal dysbiosis, epithelial findings are not strictly dichotomous. Intermediate and mixed presentations may occur, particularly in women with overlapping microbiological patterns or Nugent intermediate scores. Epithelial alterations should therefore be interpreted within the broader microbiological and clinical context rather than as isolated disease-specific markers.

### 4.6. Clinical Consequences

Both BV and AV have been associated with adverse reproductive and obstetric outcomes, but the mechanisms through which these outcomes arise appear to differ.

BV has consistently been linked to preterm birth, pelvic inflammatory disease, acquisition of sexually transmitted infections, and postoperative infectious complications [29,46,47]. These risks are generally attributed to ecological disruption characterized by loss of lactobacillary dominance, increased microbial diversity, and persistence of anaerobic biofilm communities capable of ascending infection.

AV has also been associated with preterm birth, chorioamnionitis, and neonatal infections [45,48,49]. In this setting, the inflammatory component appears particularly important. Neutrophil recruitment, epithelial damage, and local immune activation may contribute directly to tissue injury [12,49].

Importantly, adverse outcomes are not confined to women who fit neatly into classical BV or AV categories. Intermediate and overlapping microbial states, especially those corresponding to Nugent scores of 4–6, may also carry clinically relevant risks, although these risks appear heterogeneous and less predictable. This variability likely reflects the coexistence of multiple underlying mechanisms within the intermediate group, including biofilm-associated anaerobic dysbiosis and inflammation-dominant aerobic patterns. To further complicate the clinical interpretation of BV and AV, most major randomized clinical trials in pregnancy have grouped intermediate Nugent scores together with overt BV and have not considered AV at all, thereby introducing substantial confounding and significant selection bias in these studies [50].

However, from a clinical perspective, similar reproductive outcomes may arise through different pathobiological pathways. This reinforces the concept that vaginal dysbiosis is biologically heterogeneous, even when clinical outcomes appear superficially similar.

### 4.7. Aerobic Dysbiosis Beyond Classical Entities and the Spectrum of Inflammatory Vaginal States

The concept of vaginal dysbiosis extends beyond the classical dichotomy of BV and AV. Increasing evidence suggests that aerobic inflammatory vaginal phenotypes can arise in a variety of physiological, transitional, and pathological contexts that are only partially captured by current diagnostic frameworks.

In this broader perspective, the AV-like phenotype should not be viewed exclusively as a discrete disease entity. Rather, it may represent a context-dependent ecological response resulting from the interaction between microbial communities, host immunity, epithelial integrity, and endocrine status.

The puerperium provides a clear example. Abrupt postpartum hormonal withdrawal leads to reduced estrogen levels, increased vaginal pH, transient lactobacillary depletion, colonization by mixed aerobic flora, leukocyte recruitment, and epithelial remodeling. Although these findings may fulfill several criteria used in AV scoring systems, their self-limited nature and close relationship to physiological recovery argue against interpreting all such cases as pathological AV.

A similar overlap is observed in hypoestrogenic states such as postmenopause. Long-term estrogen deficiency promotes lactobacillary reduction, epithelial fragility, and variable colonization by aerobic organisms. AV-like microbiological patterns may persist, but the associated inflammatory component is often mild or inconsistent and may reflect atrophic rather than infectious mechanisms.

Post-antibiotic states provide another example. Following abrupt microbiota depletion, opportunistic aerobic species may transiently predominate, often accompanied by nonspecific inflammatory changes. These patterns are frequently reversible and depend on subsequent microbiome reconstitution.

Collectively, these observations indicate that AV-like inflammatory vaginal phenotypes should be interpreted within their physiological and clinical context rather than automatically classified as a single disease process. The same microscopic pattern may correspond to infectious inflammation, hormonal adaptation, tissue repair, or transitional ecological recovery.

## 5. The Influence of Hormonal Status and Life Stages

Hormonal status is one of the major physiological determinants of the vaginal ecosystem and profoundly influences microbial composition, epithelial maturation, and mucosal immune responses throughout a woman’s life. Because current diagnostic criteria for vaginal dysbiosis have largely been developed in women of reproductive age, their interpretation may become problematic when applied to physiological conditions characterized by different endocrine environments. As a result, identical microscopic findings may reflect fundamentally different biological processes depending on the patient’s hormonal status.

### 5.1. Reproductive Age

The reproductive years provide the physiological framework upon which most diagnostic criteria for vaginal dysbiosis have been established. Under estrogen stimulation, the vaginal epithelium accumulates glycogen, creating an environment that favors *Lactobacillus* predominance, maintains an acidic vaginal pH, and supports epithelial homeostasis [18,51].

Within this physiological setting, depletion of lactobacilli, increased microbial diversity, epithelial disruption, or significant inflammatory infiltrates are generally interpreted as evidence of pathological dysbiosis. Accordingly, both BV and AV have traditionally been defined as deviations from this estrogen-dependent equilibrium.

Nevertheless, the vaginal ecosystem remains highly dynamic even during reproductive life. Menstrual cycle fluctuations, sexual activity, contraceptive use, and interindividual host characteristics all contribute to physiological variability in microbial composition and local immune activity [52]. These observations suggest that the concept of a single “normal” vaginal microbiota should be regarded as a useful clinical approximation rather than a rigid biological state.

### 5.2. Pregnancy, Postpartum and Lactation

Pregnancy is accompanied by profound endocrine and immunological adaptations that substantially influence the vaginal ecosystem. Elevated estrogen concentrations promote glycogen accumulation within the vaginal epithelium, favoring *Lactobacillus* dominance, maintaining low vaginal pH, and reducing overall microbial diversity compared with non-pregnant women [53,54]. These changes are generally considered protective and may contribute to reducing the risk of ascending genital tract infections during pregnancy.

Following delivery, however, the vaginal environment undergoes rapid ecological reorganization. The abrupt decline in circulating estrogen levels results in reduced epithelial glycogen content, diminished lactobacillary populations, increased vaginal pH, and transient alterations of the resident microbiota [53]. These microbial changes frequently occur together with physiological inflammatory responses associated with tissue repair and postpartum remodeling.

Lactation further prolongs this hypoestrogenic state through prolactin-mediated suppression of ovarian function. Consequently, breastfeeding women often exhibit reduced lactobacillary abundance, increased vaginal pH [55]. The resulting hypoestrogenic vaginal environment may also display microscopic features that overlap with those observed in AV despite reflecting physiological endocrine adaptation rather than an underlying infectious or inflammatory disorder.

Failure to account for postpartum and lactational physiology may therefore lead to overinterpretation of AV-like microscopic findings and, consequently, to inappropriate diagnostic or therapeutic decisions.

### 5.3. Perimenopause and Postmenopause

Perimenopause and postmenopause represent additional physiological settings in which hormonal changes profoundly influence the vaginal ecosystem. Progressive estrogen deficiency leads to reduced epithelial glycogen content, loss of *Lactobacillus* predominance, increased vaginal pH, and progressive remodeling of the vaginal microbiota [56,57].

These endocrine changes are accompanied by epithelial thinning, increased epithelial fragility, widespread presence of parabasal cells, and variable degrees of leukocyte infiltration. Clinically, women may experience vaginal dryness, burning, irritation, dyspareunia, and mucosal fragility, manifestations that substantially overlap with those commonly attributed to AV [40].

Distinguishing AV from genitourinary syndrome of menopause may therefore be challenging. Although genuine AV certainly occurs in postmenopausal women, many microscopic features traditionally interpreted as pathological in reproductive-age patients may instead represent physiological consequences of estrogen deficiency. This physiological overlap becomes particularly relevant when interpreting intermediate or atypical microscopic findings and may contribute to the biological heterogeneity observed within current diagnostic classifications.

### 5.4. Interpreting Microscopic Findings According to Hormonal Milieu

Several microscopic features incorporated into current diagnostic algorithms are strongly influenced by hormonal status and should therefore be interpreted within the appropriate physiological context.

Lactobacillary depletion is widely regarded as a hallmark of BV and an indicator of vaginal dysbiosis. However, reduced *Lactobacillus* abundance also occurs physiologically during postpartum, lactation, and menopause, where it may reflect endocrine adaptation rather than pathological microbial overgrowth [53,54,55].

Similarly, parabasal epithelial cells represent an important component of the AV scoring system because they indicate epithelial regeneration and mucosal injury [10]. Yet these cells are also commonly observed in hypoestrogenic conditions, where they primarily reflect physiological epithelial atrophy rather than inflammation-driven epithelial damage.

Interpretation of inflammatory findings presents comparable challenges. Although abundant leukocytes are characteristic features of AV, physiological fluctuations in local immune activity occur throughout the menstrual cycle and during pregnancy, postpartum, and postmenopause [53,54,55,56,57]. Consequently, distinguishing physiological immune activation from pathological inflammation is not always straightforward and requires careful integration of microscopic findings with clinical and hormonal context.

**Figure 3:**
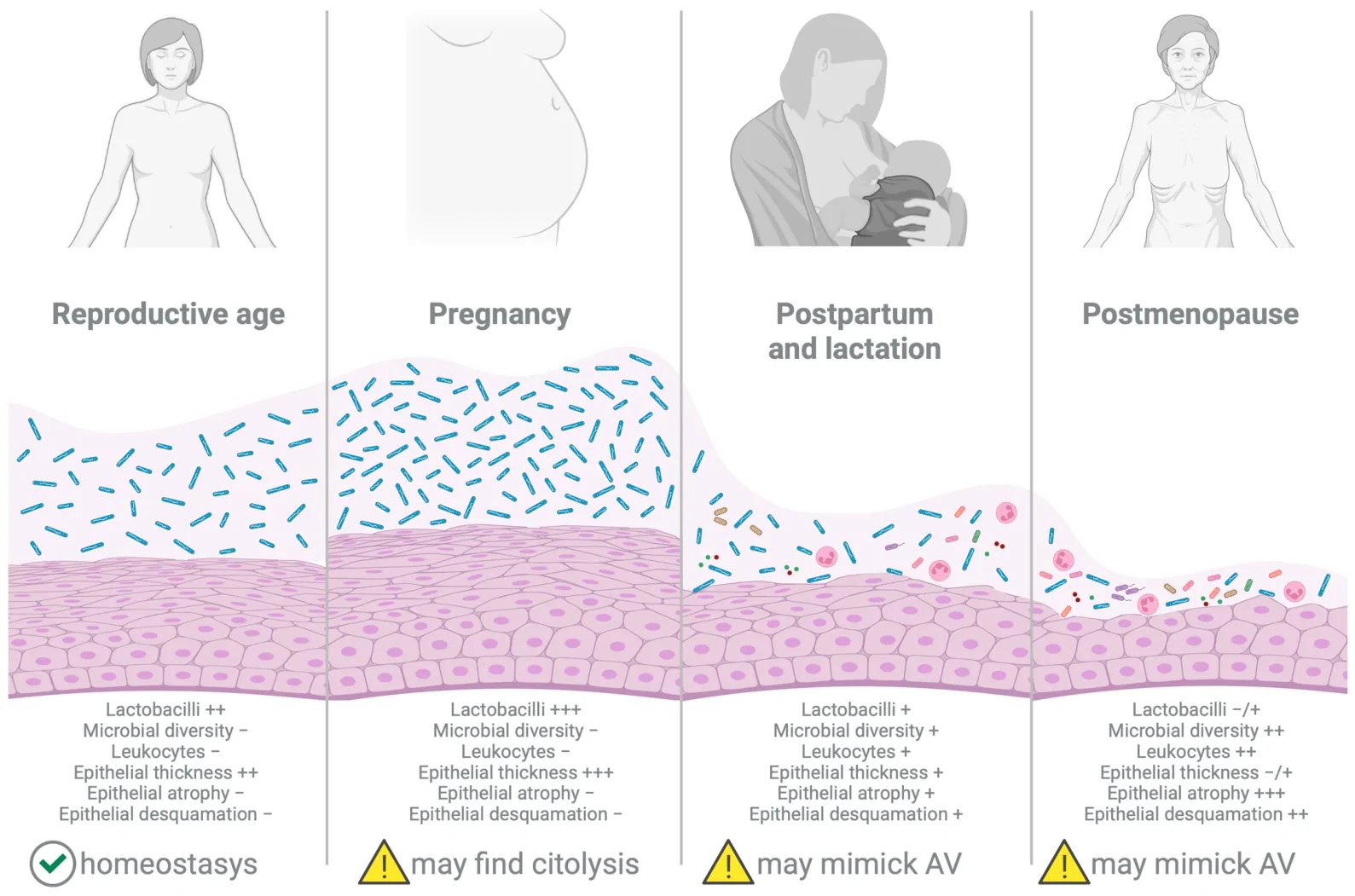
Influence of hormonal status on the interpretation of vaginal microbiological patterns. Conceptual framework illustrating how hormonal milieu modulates vaginal microbiota composition and the interpretation of microscopic findings across different life stages, including reproductive age, pregnancy, postpartum period, lactation, perimenopause, and menopause. The figure emphasizes that similar microbiological patterns may correspond to distinct clinical and inflammatory states depending on estrogen levels, epithelial maturation, and host immune response, thereby contributing to variability in the expression of both bacterial vaginosis and aerobic vaginitis phenotypes. Created in https://BioRender.com.

Overall, hormonal status substantially modifies the biological meaning of many microscopic findings that are commonly incorporated into diagnostic algorithms. Similar cytological or microbiological patterns may therefore represent fundamentally different physiological or pathological conditions depending on the endocrine context in which they occur. This observation reinforces the need to interpret vaginal microscopy within the broader clinical and physiological setting rather than as an isolated diagnostic test (Figure 3).

## 6. Diagnostic Approaches and Competing Paradigms

The diagnosis of vaginal dysbiosis remains challenging because currently available diagnostic approaches are founded on different conceptual frameworks and interrogate distinct biological domains. Some methods primarily characterize microbial composition, whereas others incorporate inflammatory response, epithelial alterations, or clinical manifestations. Consequently, discordant diagnostic results are common, particularly in women presenting with intermediate, mixed, or otherwise uncommon vaginal ecosystems. Understanding both the strengths and the limitations of each diagnostic paradigm is therefore essential for the appropriate interpretation of vaginal disorders.

### 6.1. Clinical Diagnosis

Clinical diagnosis of BV traditionally relies on the Amsel criteria, which include homogeneous vaginal discharge, elevated vaginal pH (>4.5), a positive amine (“whiff”) test, and the presence of clue cells on wet mount microscopy. The presence of at least three of these four criteria is generally considered diagnostic of BV [8].

The Amsel criteria remain widely used because they are inexpensive, rapidly applicable in routine practice, and require minimal laboratory resources. However, their interpretation is inherently subjective and depends considerably on examiner experience [8]. Moreover, they were specifically developed to identify classical BV and may perform less well in women presenting with intermediate or mixed dysbiotic patterns.

By contrast, no universally accepted clinical criteria exist for AV. Typical manifestations, including yellow discharge, vaginal irritation, burning, dyspareunia, and mucosal erythema, are non-specific and frequently overlap with those observed in vulvovaginal candidiasis, trichomoniasis, genitourinary syndrome of menopause, and other inflammatory disorders. Clinical examination alone is therefore insufficient to establish a reliable diagnosis of AV and should be complemented by microscopic assessment [40].

### 6.2. Microscopy-Based Diagnosis

Microscopy remains the cornerstone of vaginal microbiological assessment because it simultaneously provides information on microbial composition, inflammatory response, and epithelial status [26,58]. Unlike purely microbiological techniques, it allows direct visualization of the interaction between microorganisms and host tissues, thereby offering a more comprehensive evaluation of the vaginal ecosystem.

Although the Amsel criteria include one microscopic parameter, the identification of clue cells, they were primarily designed for the diagnosis of BV and provide little information regarding inflammation or epithelial injury.

The Gramstained-based scoring system proposed by Nugent et al. [9] remains the most widely accepted laboratory reference method for BV diagnosis. This scoring system quantifies bacterial morphotypes associated with lactobacilli, *Gardnerella* morphotypes, and curved Gram-variable rods (often referred to as *Mobiluncus*), categorizing specimens as normal (0–3), intermediate (4–6), or BV (7–10). However, Nugent scoring evaluates bacterial morphotypes exclusively and provides no information regarding host inflammatory response, epithelial alterations, or the presence of aerobic microorganisms. Consequently, inflammatory disorders such as AV may remain unrecognized or be incorporated into the intermediate category.

An alternative Gram stain-based classification, the Ison–Hay classification proposed by Ison and Hay [59], was subsequently proposed to simplify microscopic assessment by categorizing vaginal flora into qualitative grades according to the relative predominance of *Lactobacillus* morphotypes and mixed bacterial morphotypes. Although less granular than the Nugent score, it has been widely adopted in clinical practice and reflects the same underlying concept of classifying vaginal dysbiosis primarily according to bacterial composition.

The Lactobacillary Grade (LBG) system, also introduced by Donders et al. [10], was developed to characterize the relative abundance of lactobacilli on wet mount microscopy and to provide a more ecological description of the vaginal flora. Although valuable for describing alterations in lactobacillary dominance, LBG does not incorporate inflammatory parameters and therefore cannot reliably distinguish between inflammatory and non-inflammatory forms of dysbiosis.

The AV scoring system proposed by Donders et al. [10] represented a major conceptual advance by integrating microbial and host-related variables within a single microscopic assessment. In addition to lactobacillary depletion and background flora, it evaluates leukocyte abundance, toxic leukocytes, and parabasal epithelial cells, thereby incorporating inflammatory and epithelial dimensions into the diagnostic process. Nevertheless, several limitations remain. Interpretation of some parameters is subjective, interobserver variability may be substantial, and mixed anaerobic-aerobic communities are not always readily classified [19,37]. Furthermore, physiological conditions such as postmenopause and the puerperium may generate AV-like microscopic appearances, potentially reducing diagnostic specificity.

Thus, meaningful interpretation of vaginal microscopy requires integration of multiple microscopic features rather than isolated assessment of individual parameters.

### 6.3. Cultural and Molecular Diagnostics

Culturing microorganisms from vaginal swabs allows identification of species even when they are present in low abundance, thanks to selective culture media. This is both the main advantage and the main limitation of the technique, as it requires specific media and culture conditions for each target microorganism. Nevertheless, culture remains the preferred method to confirm vaginal candidiasis when symptoms are suggestive but microscopy is negative.

Molecular methods, including NAATs based on quantitative or multiplex PCR, as well as NGS, have substantially expanded current knowledge of the vaginal microbiome. Commercial molecular assays demonstrate excellent diagnostic performance for BV and frequently outperform conventional microscopy with respect to analytical sensitivity and reproducibility [60,61,62]. In parallel, sequencing studies have revealed the remarkable diversity and dynamic nature of vaginal microbial communities, challenging the traditional dichotomous distinction between health and disease.

Despite these advantages, molecular diagnostics also present important limitations. Current assays are designed to characterize microbial composition and provide no direct information regarding host inflammatory response, epithelial integrity, leukocyte activity, or biofilm architecture. Consequently, identification of microorganisms potentially associated with dysbiosis does not necessarily indicate clinically relevant disease. Likewise, molecular methods may detect dysbiotic microbial signatures in asymptomatic women or fail to distinguish active disease from colonization or transient ecological variation. Furthermore, interpretation of molecular assays also depends on the biological context in which they were developed and validated. This issue has recently been highlighted in transgender populations, where neovaginal anatomy or testosterone-induced changes in the vaginal ecosystem may reduce the applicability of diagnostic algorithms originally established for cisgender women [63].

Accordingly, molecular diagnostics should complement rather than replace clinical evaluation and microscopy.

### 6.4. Why Diagnostic Systems May Disagree

Disagreement among diagnostic methods should not be regarded as unexpected, since each approach examines different components of the vaginal ecosystem. Clinical criteria primarily assess symptoms and physical signs, Nugent and Ison–Hay scoring evaluate bacterial morphotypes, AV scoring incorporates inflammatory and epithelial parameters, whereas molecular techniques characterize microbial composition with high analytical sensitivity.

As a consequence, the same patient may receive different diagnostic classifications depending on the method employed. Women with inflammatory aerobic dysbiosis, for example, may exhibit intermediate Nugent scores despite fulfilling microscopic criteria for AV. Conversely, molecular assays may identify BV-associated anaerobes in women who lack both clinical manifestations and microscopic evidence of disease. Diagnostic discordance is further increased by mixed vaginitis, post-treatment microbiota reconstitution, and physiological hypoestrogenic states.

Rather than indicating that one diagnostic method is necessarily incorrect, these discrepancies illustrate the complex nature of vaginal dysbiosis. Each diagnostic framework captures only part of the underlying biological intricacy, and apparent disagreement often reflects the fact that different methods interrogate different, only partially overlapping, components of the vaginal ecosystem.

## 7. International Guidelines: Do Current Recommendations Reflect Biological Reality?

The growing recognition of the vaginal ecosystem as a dynamic interplay between microbial communities, host immunity, epithelial integrity, and endocrine status raises important questions regarding the adequacy of current diagnostic recommendations. Although international guidelines have substantially improved the standardization of vaginitis diagnosis and management, they have largely evolved within conceptual frameworks originally developed for classical BV and other pathogen-oriented conditions. Whether these recommendations fully reflect the biological heterogeneity of vaginal dysbiosis remains an open question.

### 7.1. BV in International Guidelines: Diagnostic Hierarchy and Standardization

Major international guidelines, including those issued by the Centers for Disease Control and Prevention (CDC), the International Union against Sexually Transmitted Infections (IUSTI), and the International Society for the Study of Vulvovaginal Disease (ISSVD), recognize BV as a distinct clinical entity characterized by depletion of lactobacilli and overgrowth of anaerobic microorganisms [13,14,15]. Across these documents, diagnosis is primarily based on the Amsel clinical criteria and, particularly in research settings, on Nugent scoring, which remains the reference laboratory method for BV diagnosis.

The widespread adoption of standardized diagnostic criteria has greatly facilitated comparisons among clinical studies and contributed to relatively uniform management strategies worldwide. Nevertheless, these diagnostic frameworks were specifically developed to identify classical anaerobic dysbiosis and therefore may not encompass the full spectrum of vaginal ecological disturbances encountered in routine practice.

Although molecular assays have demonstrated excellent analytical performance for BV, their role within current guidelines remains complementary, and they have not replaced microscopy-based diagnostic standards [13,14,15].

### 7.2. Beyond BV: Heterogeneous Recognition of Non-Classical Vaginal Dysbiosis

Recognition of other forms of vaginal dysbiosis is considerably less consistent across international recommendations. AV, despite increasing scientific interest and a growing body of evidence supporting its clinical relevance [40], is not recognized as a distinct diagnostic entity in the current CDC guidelines [13]. By contrast, several European expert documents and microscopy-oriented recommendations acknowledge AV and consider wet mount microscopy the reference diagnostic method [14,15].

Similarly, DIV remains incompletely incorporated into standardized diagnostic algorithms, reflecting both ongoing debate regarding its relationship with AV and the absence of universally accepted diagnostic criteria [27,28].

Cytolytic vaginosis provides another illustrative example. This condition is characterized by excessive lactobacillary proliferation, epithelial cytolysis, and symptoms that frequently mimic vulvovaginal candidiasis. Its recognition varies substantially across international recommendations, highlighting the broader challenge of incorporating conditions that are not defined by a specific pathogen into conventional diagnostic frameworks. Unlike infectious vaginitis, diagnosis relies primarily on recognition of a characteristic microscopic pattern rather than identification of a causative microorganism.

Collectively, these observations indicate that current recommendations predominantly address disorders defined by characteristic microbial signatures, whereas conditions arising from more complex interactions among microbial communities, epithelial alterations, and host inflammatory responses remain less consistently represented.

### 7.3. Wet Mount Microscopy as a Pattern-Based Diagnostic Paradigm

The variable recognition of AV, DIV, and cytolytic vaginosis reflects a more fundamental issue in the diagnosis of vaginal disorders. Unlike BV, these conditions cannot be adequately characterized solely through identification of specific microorganisms. Their diagnosis instead depends on integrated interpretation of multiple microscopic features, including inflammatory response, epithelial alterations, cytolysis, background microbiota, and, in selected clinical settings, hormonal status.

Among currently available diagnostic techniques, wet mount microscopy uniquely provides simultaneous assessment of all these biological domains and therefore remains the reference diagnostic method for conditions such as AV, while also representing the only technique capable of directly identifying cytolysis, whose clinical significance remains debated. Neither culture-based methods nor currently available molecular assays can directly evaluate leukocyte activity, epithelial injury, toxic leukocytes, cytolysis, or other dynamic host-related features that are fundamental to the recognition of these disorders, although the clinical significance and diagnostic criteria of cytolytic vaginosis remain incompletely defined [64].

Diagnostic strategies based exclusively on microbial identification may therefore prove insufficient whenever disease expression depends less on the presence of a particular microorganism than on the interaction between microbial communities and host tissues. This distinction may partly explain why some vaginal disorders integrate readily into guideline-based diagnostic algorithms, whereas others continue to challenge conventional classification systems.

Overall, current international recommendations provide a robust framework for the diagnosis and management of classical BV. At the same time, accumulating evidence suggests that the biological diversity of vaginal dysbiosis extends beyond pathogen-centered models, indicating that existing diagnostic frameworks capture only part of the complexity of the vaginal ecosystem.

## 8. The Grey Zones: Intermediate, Mixed and Transitional States

Increasing evidence indicates that vaginal dysbiosis cannot always be categorized into discrete and mutually exclusive diagnostic entities. In routine clinical practice, a substantial proportion of women exhibit microbiological and inflammatory patterns that do not fully satisfy the diagnostic criteria for either classical BV or AV. These intermediate, mixed, transitional, and uncommon states challenge traditional classification systems and suggest that vaginal dysbiosis may be better conceptualized as a dynamic spectrum rather than as a collection of distinct diseases.

### 8.1. Intermediate Flora

The intermediate Nugent score category (score 4–6) has traditionally been interpreted as a transitional state between eubiosis and BV [65]. However, accumulating evidence suggests that this interpretation may be overly simplistic. Women classified as having intermediate flora constitute a biologically heterogeneous group exhibiting diverse microbial, inflammatory, and clinical profiles.

Some individuals with intermediate Nugent scores may indeed represent incipient or resolving BV, whereas others may harbor inflammatory conditions consistent with AV, mixed anaerobic–aerobic dysbiosis, physiological hypoestrogenic states, post-treatment microbiota reconstitution, or low-load microbial communities. Furthermore, molecular studies have demonstrated considerable heterogeneity within intermediate flora, revealing microbial compositions that cannot be readily assigned to either healthy *Lactobacillus*-dominated states or classical BV-associated communities [66].

The recognition of AV has further challenged the traditional interpretation of intermediate flora. Because Nugent scoring exclusively evaluates bacterial morphotypes and does not assess inflammation or epithelial alterations, inflammatory dysbiosis may remain undetected and be categorized within the intermediate range. Consequently, at least a subset of women currently classified as having intermediate flora may not represent transitional dysbiosis, but rather biologically distinct inflammatory phenotypes.

These observations suggest that the intermediate Nugent category should not be regarded as a single diagnostic entity, but rather as a heterogeneous umbrella encompassing multiple biological states with potentially different clinical implications (Table 1).

### 8.2. BV and AV Overlap

BV and AV are traditionally considered distinct entities characterized by fundamentally different microbiological and immunological features. Nevertheless, increasing clinical experience indicates that overlap between anaerobic dysbiosis and inflammatory vaginitis is not uncommon [19].

Women may simultaneously exhibit depletion of lactobacilli, overgrowth of anaerobic BV-associated bacteria, and microscopic evidence of inflammation, including abundant leukocytes, and epithelial disruption. Such cases cannot be easily classified using conventional diagnostic frameworks and frequently generate discordant results between Nugent scoring, AV scoring, and molecular assays.

Several mechanisms may contribute to this overlap. Anaerobic dysbiosis itself may induce inflammatory responses in some women, whereas secondary colonization by aerobic microorganisms may occur in pre-existing BV-associated ecological disturbances. Host-related factors, including genetic susceptibility, immune response, hormonal status, and epithelial barrier integrity, may further modulate disease expression.

Rather than representing exceptional situations, these overlap patterns suggest that the boundaries between BV and AV are not always sharply defined. They reinforce the concept that the vaginal ecosystem may express combined microbial and inflammatory phenotypes that cannot be fully accommodated within conventional dichotomous classifications.

### 8.3. Mixed Vaginitis

Mixed vaginitis refers to the simultaneous presence of at least two vaginal disorders contributing to the patient’s symptoms and clinical manifestations [37]. Importantly, mixed vaginitis should be distinguished from asymptomatic microbial coexistence, since the mere detection of multiple microorganisms does not necessarily imply pathogenic interaction.

The most frequently reported mixed presentations include BV combined with vulvovaginal candidiasis, BV associated with AV, AV associated with candidiasis, and trichomoniasis accompanied by additional dysbiotic conditions [67,68]. These combinations often present with overlapping symptoms and complex microscopic findings, complicating both diagnosis and treatment.

BV and candidiasis frequently coexist despite their apparently contrasting ecological characteristics. Similarly, AV may coexist with candidiasis, particularly in women with epithelial disruption and inflammatory changes. Trichomoniasis may further modify the vaginal ecosystem by inducing intense inflammation and facilitating secondary microbial alterations.

The recognition of mixed vaginitis has important therapeutic implications because management directed at only one component may fail to achieve complete resolution. Consequently, comprehensive microscopic assessment remains essential for identifying concomitant pathological processes which may also be confirmed using cultural-based methods or molecular assays.

### 8.4. Transitional and Post-Treatment States

Vaginal microbial communities are highly dynamic and may undergo substantial changes during treatment, recovery, and physiological transitions [69,70,71,72]. Following antimicrobial therapy, many women exhibit intermediate or uncommon microbial profiles that cannot be readily classified according to conventional diagnostic criteria.

Post-treatment states may be characterized by partial restoration of lactobacilli, persistence of residual dysbiotic communities, transient inflammatory responses, or marked reductions in overall microbial load. Similar transitional patterns may occur during menstruation, pregnancy, postpartum hypoestrogenism, and menopausal transition.

These observations emphasize that vaginal dysbiosis should not be viewed as a static condition. Rather, vaginal microbial ecosystems continuously evolve under the influence of host physiology, environmental exposures, and therapeutic interventions. Sexual activity may further contribute to transient ecological fluctuations through semen exposure and microbial exchange between partners, occasionally generating intermediate or less common vaginal patterns [73,74,75].

### 8.5. Non-Classifiable and Uncommon Microscopic Patterns

Routine microscopy frequently reveals vaginal patterns that do not conform to established diagnostic categories. Such findings may include severe lactobacillary depletion in the absence of obvious pathogens, abundant coccoid flora without inflammation, pronounced inflammation without identifiable microorganisms, low-load communities, or discordant inflammatory and microbiological features.

In some cases, these uncommon patterns may reflect technical limitations or transient ecological fluctuations. In others, they may represent currently unrecognized biological phenotypes that are not adequately captured by existing classification systems.

The increasing application of molecular techniques has further highlighted the existence of discordant cases in which microbial signatures identified by sequencing or NAATs do not correspond to clinical or microscopic findings. Such discrepancies reinforce the concept that current diagnostic categories may only partially reflect the complexity of vaginal ecosystem disturbances.

### 8.6. Cytolytic Vaginosis as a Diagnostic Challenge

Cytolytic vaginosis has been proposed as a paradigmatic example of a non-classical vaginal disorder that challenges pathogen-centered diagnostic models. The condition has been described as being characterized by excessive proliferation of lactobacilli, extensive epithelial cytolysis, low vaginal pH, and symptoms frequently resembling vulvovaginal candidiasis, including pruritus, burning, and dyspareunia [76].

Unlike infectious vaginitis, cytolytic vaginosis is not associated with a specific pathogen. When cytolytic vaginosis is suspected, diagnosis has traditionally relied on the recognition of characteristic microscopic findings, including abundant lactobacilli, epithelial cell lysis, paucity of leukocytes, and the absence of fungal elements or other pathogens [76].

Because neither culture-based methods nor currently available molecular assays can adequately capture these cytological alterations, wet mount microscopy remains essential for diagnosis. If accepted as a distinct clinical entity, cytolytic vaginosis would exemplify how vaginal symptoms may arise not only from pathogen overgrowth but also from alterations in host–microbiota interactions. Furthermore, cytolytic changes may overlap with other dysbiotic or inflammatory conditions, particularly during transitional or post-treatment states, thereby further increasing diagnostic complexity.

Overall, intermediate, mixed, transitional, and uncommon vaginal states highlight the limitations of rigid dichotomous classifications and support the concept that vaginal dysbiosis is better understood as a dynamic continuum of interacting microbial and host-related processes.

## 9. Are BV and AV Mutually Exclusive Diseases?

### 9.1. The Strengths of the Traditional Dichotomous Paradigm

Since their original descriptions, BV and AV have been regarded as distinct clinicopathological entities characterized by different microbial communities, microscopic features, and host responses. Classical BV is typically associated with anaerobic dysbiosis, polymicrobial biofilm formation, and a relatively limited inflammatory response, whereas AV is characterized by depletion of lactobacilli, predominance of aerobic or facultative microorganisms, epithelial disruption, and marked inflammation [29,40].

This distinction has provided substantial conceptual and clinical advantages. Separate diagnostic criteria have facilitated standardization in clinical practice and research, improved communication among clinicians, and enabled more consistent evaluation of epidemiological associations, therapeutic interventions, and reproductive outcomes. Equally important, recognition of AV as a condition distinct from BV has drawn attention to the diagnostic relevance of host inflammatory response and epithelial alterations, biological dimensions that are only marginally addressed by conventional BV-oriented diagnostic systems.

Overall, the available evidence supports the view that BV and AV represent biologically distinct conditions rather than different manifestations of a single disease process.

### 9.2. Observations Challenging Strict Mutual Exclusivity

Despite the usefulness of this traditional dichotomous framework, an increasing number of clinical and biological observations indicate that the distinction between BV and AV is not always straightforward.

As discussed in previous sections, the intermediate Nugent score category encompasses biologically heterogeneous conditions that may include inflammatory phenotypes, mixed dysbiosis, physiological hypoestrogenic states, and post-treatment microbiota reconstitution. Likewise, overlap between anaerobic dysbiosis and inflammatory vaginitis is frequently encountered in routine practice, with some women simultaneously exhibiting microbiological and microscopic features consistent with both BV and AV.

The existence of mixed vaginitis, transitional ecological states, and uncommon microscopic patterns further complicates the distinction between these disorders. In addition, accumulating evidence indicates that host-related factors—including immune response, epithelial integrity, hormonal status, and environmental influences—may substantially modulate disease expression, generating different clinical phenotypes despite partially similar microbial compositions.

Taken together, these observations indicate that, although BV and AV can generally be recognized as distinct clinicopathological entities, a strictly dichotomous classification does not adequately accommodate the full range of biological and clinical findings encountered in routine practice.

### 9.3. An Unresolved Biological Question

Current evidence strongly supports the biological distinction between BV and AV, and the traditional dichotomous framework has undoubtedly provided major diagnostic, therapeutic, and research advantages.

Taken together, the evidence reviewed in this manuscript indicates that the classical BV/AV dichotomy remains clinically useful but does not fully capture the biological diversity of vaginal ecosystem states. Rather than invalidating the concepts of BV and AV, these observations indicate that they should be regarded as useful clinicopathological constructs that describe two of the predominant and best-characterized vaginal dysbiosis phenotypes within a much broader spectrum of vaginal ecosystem states. Their apparent mutual exclusivity therefore likely reflects a necessary diagnostic simplification rather than the full biological diversity of vaginal ecosystem states.

Instead of consisting of discrete disease states separated by clear biological boundaries, the vaginal ecosystem appears to comprise dynamic and interacting microbial, inflammatory, epithelial, hormonal, and ecological processes whose boundaries, temporal evolution, and biological significance remain only partially understood.

## 10. Strength and Limitations

This review has several strengths. It provides an integrated overview of the historical evolution, biological basis, diagnostic approaches, and current classification challenges of BV and AV, bringing together evidence that is usually discussed separately. By focusing on the biological and ecological interpretation of vaginal dysbiosis, it also highlights areas where current diagnostic frameworks may not fully reflect the complexity of the vaginal ecosystem.

This review also has limitations. As a narrative review, it does not follow the methodology of a systematic review and no formal assessment of study quality or certainty of evidence (e.g., GRADE) was performed. The conclusions therefore reflect a critical synthesis of the available literature rather than a quantitative evaluation of the evidence. In addition, some of the conceptual interpretations discussed represent hypotheses intended to stimulate further investigation rather than validated diagnostic models.

## 11. Conclusions

Bacterial vaginosis and aerobic vaginitis have become well-established clinicopathological entities over the past decades, each characterized by distinctive microbiological, microscopic, and clinical features. This distinction has substantially advanced the understanding of vaginal dysbiosis by providing standardized diagnostic criteria, improving communication among clinicians, and supporting more consistent clinical management and research.

The evidence reviewed in this article indicates that current diagnostic classifications, although clinically valuable, do not fully capture the biological diversity of vaginal ecosystem states. Consequently, the central question is no longer whether BV and AV represent distinct biological conditions, as the available evidence strongly supports this distinction. The more relevant question is whether the current diagnostic frameworks adequately capture the diversity of vaginal ecosystem states encountered in everyday clinical practice. Although existing classifications remain highly useful, the evidence reviewed in this article indicates that they inevitably simplify a biological system whose ecological interactions are far more dynamic and heterogeneous than current diagnostic categories can fully encompass. Addressing this complexity will require moving beyond the interpretation of isolated microorganisms or individual diagnostic scores toward a broader appreciation of the interactions between microbial communities, host inflammatory responses, epithelial integrity, hormonal milieu, and environmental influences. Characterizing these interactions, together with their temporal evolution, represents one of the major challenges for future research and may ultimately lead to a more biologically grounded interpretation of vaginal dysbiosis while preserving the clinical utility of existing diagnostic frameworks. Such advances may also support more individualized clinical management by improving the biological characterization of vaginal ecosystem states.

## Figures and Tables

**Table 1:** Hypothesized ^1^ Biological Contributors to Intermediate Nugent Scores.

Hypothesized Contributor	Possible Microscopic Features Contributing to an Intermediate Nugent Score
Possible early BV-related dysbiosis	Lactobacilli reduction with increasing BV-associated morphotypes
Possible AV phenotype	Lactobacilli reduction with inflammatory findings (e.g., leukocytes and parabasal cells) identified by dedicated AV assessment
Mixed BV-AV	Features consistent with both BV and AV when independently evaluated
Hypoestrogenic state ^2^	Lactobacilli reduction, widespread parabasal cells and variable inflammatory changes
Post-antibiotic	Low bacterial biomass with reduced lactobacilli and background bacteria
Indeterminate	Minimal lactobacilli reduction with minor nonspecific bacterial morphotypes

Abbreviations: BV, bacterial vaginosis; AV, aerobic vaginitis. ^1^ The conditions listed are not intended as validated subcategories of intermediate Nugent flora, but as hypothetical biological scenarios that may contribute to intermediate Nugent scores. Importantly, AV and mixed BV–AV cannot be diagnosed from the Nugent score alone and require independent evaluation using dedicated microscopic criteria. The scenarios shown are conceptual hypotheses derived from the biological interpretation of the available literature discussed throughout this review and are not intended to represent validated diagnostic categories. ^2^ Physiological hypoestrogenic states are included as non-pathological conditions that may contribute to intermediate Nugent scores when interpreted outside their clinical context.

## Data Availability

No new data were created or analyzed in this study. Data sharing is not applicable to this article.

## Abbreviations

The following abbreviations are used in this manuscript:

AV	Aerobic Vaginitis
BV	Bacterial Vaginosis
CDC	Centers for Disease Control and Prevention
DIV	Desquamative Inflammatory Vaginitis
ISSVD	International Society for the Study of Vulvovaginal Disease
IUSTI	International Union against Sexually Transmitted Infections
LBG	Lactobacillary Grade
NAATs	Nucleic Acid Amplification Tests
NGS	Next Generation Sequencing
PCR	Polymerase Chain Reaction
